# Exploring Adjustment and Parent–Infant Relations in Mothers of Premature Infants: Thematic Analysis Using a Multisensory Approach

**DOI:** 10.1093/jpepsy/jsac007

**Published:** 2022-03-14

**Authors:** Catalina Suarez, Pauline Adair, Nicola Doherty, David McCormack

**Affiliations:** Department of Psychology, Queen’s University Belfast, Belfast, Northern Ireland; Department of Psychology, Queen’s University Belfast, Belfast, Northern Ireland; Western Health and Social Care Trust, Londonderry, Northern Ireland; Department of Psychology, Queen’s University Belfast, Belfast, Northern Ireland

**Keywords:** adversity, discharge from NICU, neonatal intensive care unit, NICU experiences, parental adjustment, parents of premature infants, parental wellbeing

## Abstract

**Objective:**

The aim of the study was to explore mothers’ experiences of having an infant born prematurely (28–32 weeks gestation). In particular, the study aimed to explore the developing parent–infant relationship 12–30 months since birth and the developing parental identity during hospitalization and discharge.

**Methods:**

Twelve mothers, aged between 22 and 43, participated in the semi-structured interviews. The mean age of infants was 19 months. Interviews comprised open-ended questions and visual stimuli consisting of photographs brought by participants, word selection, and card sorting techniques. Data were analyzed using Braun and Clarke’s thematic analysis (Braun & Clarke, 2013).

**Results:**

Three themes arose from a clustering of 10 subthemes: (a) Emotional Impact, (b) Searching for Parent Identity, and (c) Moving Beyond Adversity. Participants expressed experiencing heightened emotional distress during the time of their infants’ birth and hospitalization and initially not feeling like parents. Their parental identity strengthened as they became more involved in the care of their infant and began to accept the situation. Participants described parenting their premature infants differently compared with parents of full-term infants, and described adjusting to this difference over time.

**Conclusions:**

The findings highlight the emotional experience and adjustment of mothers of premature infants, from hospital and postdischarge. The need for psycho-educational interventions postdischarge and parent-partnered models during hospitalization is discussed. In addition, the study demonstrates the use of integrating visual stimuli in qualitative data collection procedures, to elicit further meaning and interaction from participants with the interview process.

## Introduction

Premature birth is defined as infants born alive before 37 weeks gestation. Infants born between 28 and 32 weeks are considered to be “very premature” and under 28 weeks is coined “extremely premature” (World Health Organisation [WHO], 2018). Compared with full-term born infants, those born prematurely are at increased risk of illness (e.g., respiratory, cardiac, neurological, developmental), which can result in longer hospital stays, future hospital admissions, and long-term congenital conditions (Glass et al., 2015). The initial attachment relationship formed between an infant and their caregiver(s) and brain development, is dependent on the experience and interaction that the infant has with their environment, and is more sensitive to experience during the early years (Bowlby, 1988; Cassidy et al., 2013; Tierney & Nelson, 2009). Existing findings show that being born prematurely can have a negative effect on parent–infant relations as the disruption caused by early separation such as, being in a neonatal intensive care unit (NICU) for weeks postbirth, can lead to fractured bonding and differences in the dyadic interaction compared with full-term born infants (Abergel & Blicharski, 2013; Forcada-Guex et al., 2011; Geraldini, 2016). In addition, findings show that the parental identity process is impacted by the NICU experience, with parents feeling alienated, initially not feeling like a parent, and recognizing the loss of what they had expected from the birth (Hutchinson et al., 2012; Jackson et al., 2003; Rossman et al., 2015).

Several studies have focused on the psychological effects that having an infant born prematurely and spending time in the NICU has on parents. Results examining parental stress show that stress levels are higher in parents whose infants had lower gestational ages and birth weights compared with parents of full-term infants (Schappin et al., 2013), and that mothers present with higher stress levels than fathers (Carter et al., 2007). Research has also reported symptoms of post-traumatic stress disorder, depression, anxiety, and acute stress disorder in this population (Gangi et al., 2013; Helle et al., 2018; Trumello et al., 2018). A narrative synthesis of qualitative studies in peer reviewed journals found that along with the theme of stress, the disruption to the anticipated parenting role where parents described feeling helpless and not sufficiently involved in the care of their infants, was another theme commonly reported in experiences of infant NICU hospitalization (Al Maghaireh et al., 2016). According to Mercer’s theory of maternal role attainment, the maternal identity is partly formed though being involved in the care of their infant from the start which fosters self-confidence and getting to know themselves as mothers in the new relationship (Mercer, 2004). Thus, being in the NICU may hinder the maternal identity development due to the separation and limited opportunities for caregiving.

Fewer studies have focused on parents’ subjective experiences following discharge from hospital and how the transition impacts on their parent–infant relationship and identity. Holditch-Davis et al. (2015) found that mothers of infants born prematurely who at baseline reported higher levels of psychological difficulties, were at most risk of ongoing difficulties 1-year postdischarge. Other studies have shown that parents whose infants had been discharged from the NICU, consistently sought information on how to adjust to life and care for their infant (Alderdice et al., 2018; Berman et al., 2019). Hutchinson et al. (2012) have found that upon discharge, parents begin to psychologically define themselves as “caregivers” due to being less dependent on the medical system and feeling more autonomous in their parenting. Similar findings have been reported by Dellenmark-Blom & Wigert (2014) and Jackson et al. (2003). Other findings depict parents as still holding feelings of uncertainty about their parenting role and identity following discharge, with increased worries and concerns for their emotional wellbeing (Baraldi et al., 2020; White et al., 2017). These studies, albeit depicting subjective emotional experiences that parents encountered once they were home, have not focused on the experience of bonding or forming a relationship with their infants and how parents have regained and adjusted their parenting role as a result. Other limitations across previous qualitative studies are that they use diverse samples and thus include participants with clear disparities such as including parents who are not coparenting (Hutchinson et al., 2012), parents who were interviewed when their children were between 15 months and 8 years (Kantrowitz-Gordon et al., 2016), and parents with infants born at diverse gestational categories (Spinelli et al., 2016). As these disparities make it difficult to understand the perspective of a specific set of individuals, they have been addressed in the methodology of this study. Therefore, this study expands on current literature by exploring the developing parent identity/role, impact of the hospital experience on parenting, and parents’ overall experience of having an infant born prematurely from their time at hospital to postdischarge. The following questions were explored: What are primary caregivers’ experiences and memories of their time in hospital, becoming a parent in this environment, and postdischarge? What are primary caregivers’ experiences of relating to and being a parent to their premature infant?

## Methods

### Design and Materials

As the research questions focus on exploring primary caregivers’ subjective experience of their parenting journey, a qualitative research design using semi-structured interviews was adopted. The interview guide was based on the research questions and developed through consultation within the research team and reviewing previous interview questions in the area (Kantrowitz-Gordon et al., 2016; Zeanah and Benoit, 1995). The first draft was reviewed by two parents from the general population, a pediatric psychologist, and postnatal healthcare professionals. Use of innovation in qualitative research employs multisensory approaches to data collection techniques to elicit meaningful data and further understand a persons’ experience (Taylor & Coffey, 2008). This involves integrating questions in interview guides that are not only verbally asked, but also can include visual, auditory, or tactile stimuli. These questions can be the selection of words, choosing and sorting cards, and using images, music, and/or objects within the interview to elicit memories and meaning (dos Santos & Wagner, 2018; Glaw et al., 2017; Taylor & Coffey, 2008; Willig, 2017). In this study, the interview guide comprised verbal open-ended questions and visual stimuli, consisting of photographs brought by participants, a selection of words task, and a card sorting task (see Table I). These three tasks were chosen by the research team based on review of the literature, clinical experience using similar visual stimuli, and a review of a study within this area that employed photographs (Conrad & Tucker, 2019; Duara et al., 2022; Kantrowitz-Gordon et al., 2016). The team pre-empted that including questions that provided parents with visual autobiographical memory prompts (word selection, photographs) and specific parenting tasks based on child development theory (card sorting) would increase participant recall and engagement with sharing their narrative. These questions were placed at the end of sections within the interview guide to avoid impacting the fluidity of the verbal questions. Ethical approval was granted by Queens University Belfast (EPS Faculty) Research Ethics Committee.

**Table I.** Interview Schedule

*Experiences from birth/conception including NICU*
Tell me the story of your premature baby’s birth. Tell me about your feelings then and now? Tell me about a significant moment in your premature birth journey coming home from the hospital? Photographs: Why did you choose to bring these photographs? What do the images mean to you?
*Experiences following NICU*
How has it been to be a parent of a premature baby after coming home? Pick 3 words to describe your relationship with your baby. For one of these words, describe an incident or memory that illustrates what you mean. How has your relationship with your baby changed over time since birth? What is your own feeling about the change? Knowing that they were born early, do you think about them differently? If you could start all over again, knowing what you know now, what would you do differently? Divide these parent–infant activity cards into three columns: (1) Yes, *impacted by the prematurity*; (2) No, *not impacted by the prematurity*; (3) *Not Applicable*. Tell me about the cards in the “Yes” pile.^a^ When thinking of your premature birth journey so far choose two cards from each set that stand out to you. What does each phrase/word mean to you in relation to the premature birth?^b^

These 20 cards focused on parent–infant activities such as “creating and keeping routines” and “managing anxiety when with baby.” They were developed by the lead researcher from the Simms/Mann Institute Cuddlebright Experience parenting guide chapter titles and reworded in consultation with a postnatal healthcare professional.

The words used come from the ACT Conversations Values Cards developed by Dr Louise Hayes. Set 1 consisted of eight cards titled “valuing in the presence of difficulty” and set 2 consisted of eight cards titled “valuing ourselves.”

### Inclusion Criteria

Criterion sampling was used for participant recruitment. Inclusion criteria consisted of (a) primary caregivers over 18 years of age of an infant born between 28 and 32 weeks’ gestation (inclusive of infants born during 32nd week gestation); (b) at birth, infant spent time in the NICU; (c) at interview, infant was between 12- and 30-months old; and (d) infant is being co-parented. Exclusion criteria were (a) infant born as part of a multiple birth; (b) having major congenital anomalies/birth defects such as cerebral palsy, heart defects, learning disability syndromes, neural tube defects, congenital hydrocephalus; (c) main caregiver currently attending or seeking mental health services; and (d) main caregiver having more than one child born prematurely. As infants born before 28 weeks tend to be more at risk of having disabilities or congenital anomalies (Glass et al., 2015); this was the minimum gestational age chosen for the sample.

### Procedure

Participants were recruited via the social media platforms of TinyLife, a premature and vulnerable baby charity based in Northern Ireland (www.tinylife.org.uk). TinyLife provides free services within NICU’s and upon discharge to all parents living in Northern Ireland who have a premature infant and can be accessed easily by all types of families. The recruitment documentation was developed after review with TinyLife staff. An invitation to participate in the study, including a link to the information sheet, was posted at the end of May 2019 within TinyLife’s Twitter and Facebook accounts. Twelve parents contacted the research team via email expressing an interest in participating. A final round of recruitment took place at the start of August 2019 through the same means. Eight further parents contacted the research team. Telephone screening to discuss eligibility, informed consent, and the purpose of the study was conducted by the lead researcher (C.S.) who also conducted the interviews. This helped to build the relationship with potential participants prior to data collection and introduce them to the interviewer. The lead researcher was female, had no children, was employed as a Trainee Clinical Psychologist, and had previous training in research methods and data collection. Overall, five parents who met exclusion criteria were informed that they were unable to participate, and three parents did not respond to correspondence. Interviews were conducted by the lead researcher between June 6 and November 7, 2019 with parents who met inclusion criteria and who consented to participate (*n* = 12). Details of the interview, information leaflets, consent forms, and instructions regarding photographs to bring were sent to participants prior to the interview. The photograph instructions asked participants to bring two recent or past photographs that represent their premature birth journey which could include people, objects, places, or relevant images. A separate consent form was provided to participants detailing how their photographs would be used in the research and anonymized. Participants were given the option to conduct the interviews in university premises or in their homes. Five interviews took place in participants’ homes and seven took place in university premises. Within the university, interviews took place in a consistent location. Participants were asked for their infant not to be present for the interview. Prior to audio-recording, the interviews using a dictaphone, participants were asked to provide written informed consent. Participants understood that they could end their participation at any time without this affecting their care or involvement with TinyLife. Interviews were administered in an empathic style using open-ended questions (see Table I). Active listening skills were used throughout to help participants share their reflections. Prompts such as “tell me more” and “what do you mean by that” were used along with the questions to facilitate further exploration. Participants were given instructions regarding what the card sorting and word selection questions entailed and were only provided with prompts such as “tell me about the words/cards that you chose” and “what does that word mean to you?” The interviewer was vigilant for signs of emotional distress and employed the measures within the interview distress protocol which was developed in consultation with the research team's qualified clinical psychologists and guidance from Draucker et al. (2009). Upon noticing signs of distress in participants such as crying or shaking, the interview questions and recording were paused, and participants were given options regarding their participation. Seven participants exhibited signs of distress during their interview, three of which necessitated stopping the recording for a few minutes. All participants opted to continue with their interviews following this brief break, during which they regained composure. At the end of each interview, participants were provided with a debrief form containing local supports and the contact details of the clinical member of the research team. Interviews lasted an average of 65 min (range 46–89 min). Following each interview, the lead researcher notated her thoughts and emotions in a reflective research journal. Upon completion, each participant was given a CuddleBright Experience pack, which was provided by the Simms/Mann Institute free of charge (www.cuddlebright.com).

### Data Transcription and Analysis

**Figure 1.**
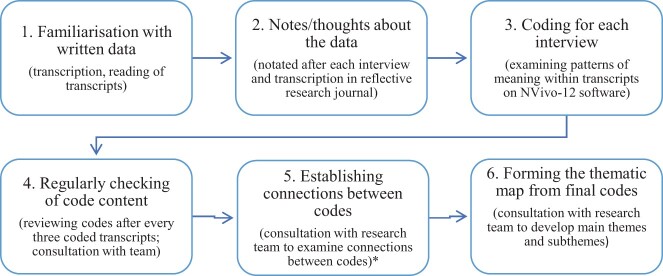
Thematic analysis six stage process. *A combination of an inductive and deductive approach was taken during stages five and six. The existing research findings in the area and research member N.D.’s clinical experience with the study population influenced the deductive approach when thinking about the code connections.

Braun and Clarke (2019) guidance on data saturation when using thematic analysis was followed, where they explain that judgments about how many interviews should be completed cannot be determined prior to analysis. Therefore the lead researcher (C.S.), while conducting the interviews reflected with the research team (P.A. and N.D.) whether new areas of discussion were arising during data collection. Following consultation, the team decided to collect no further data after 12 participants as it was felt that sufficient information was collected to answer the research questions. All interviews were transcribed verbatim by the lead researcher. Locations were anonymized and names were pseudonymized. All participants brought photographs to their interview; eight brought photographs of their infants, one participant brought photographs of objects/images, and three participants brought a mix of both. Facial features and identifying location markers within the photographs were pixelated for anonymity. Transcripts were not returned to participants for comment or correction. The data were analyzed using thematic analysis and were managed on NVivo-12 software (see Figure 1; Braun & Clarke [2006, 2013]; QSR International [n.d.]). Participants’ descriptions of the photographs they brought, and words/cards selected were analyzed and transcribed in the same way as data elicited from the verbal questions. As per the thematic analysis six stage process for analyzing data, codes were developed and reviewed for each transcript. The lead researcher conducted the analysis and reliability was obtained during stages three and four of the analysis by consulting members of the research team across three research meetings. After coding of every three transcripts, the content of each code was reviewed and refined by the lead researcher. Two additional members of the team (P.A. and N.D.) were consulted to review the codes following the initial coding of the first nine transcripts. During this consultation, the lead researcher presented the compiled direct quotes across participant transcripts for each code. This consultation resulted in renaming codes, developing more specific codes, and initial grouping of codes. Broader patterns of meaning within all transcripts were then derived from the data by the lead researcher and subsequently reviewed by the same members of the research team (P.A. and N.D.) through finding connections between the coded data (i.e., developing themes and subthemes). Final themes, subthemes, and codes were reviewed between the lead researcher and P.A., resulting in minor changes to groupings of codes. The data underlying this article are available on request from the first author.

## Results

**Figure 2.**
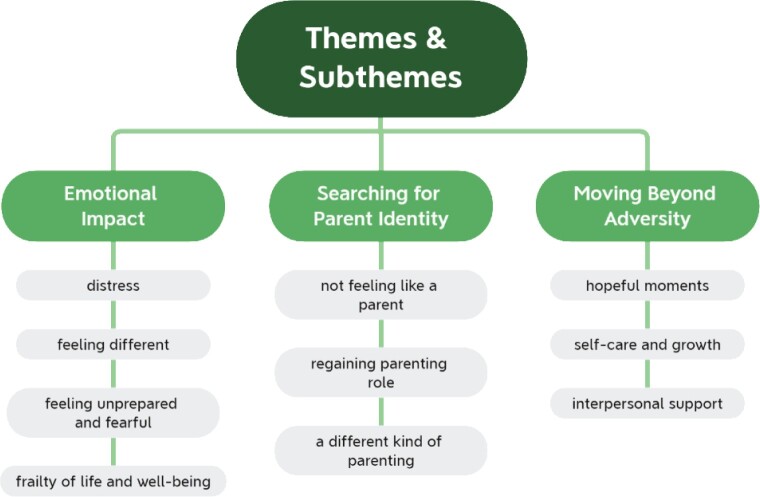
Visual map of themes and subthemes.

All 12 participants were first time mothers ranging in age between 22 and 43 years (*M *=* *32; *SD* =* *5.39). Participants were living in Northern Ireland at the time of the interviews and spoke fluent English. Their infants consisted of five females and seven males with a mean gestational age of 30 weeks (*SD* =* *1.62) and a mean hospital stay of eight weeks (*SD* =* *3.65). At interview, the infants ranged in age between 12 and 28 months (*M *=* *19; *SD* =* *5.38). Demographics are summarized in Table II. Thematic analysis of the interview transcripts led to the development of three themes (a) *Emotional Impact*, (b) *Searching for Parent Identity*, and (c) *Moving Beyond Adversity*, which arose from a clustering of 10 subthemes (see Figure 2). Themes and subthemes are discussed below, along with verbatim participant quotes and some of the photographs brought. Verbal and written consent was provided by participants to include the photographs in print form.

**Table II.** Participant Demographics

Pseudonyms gestation^a^	Parent^b^	Infant^c^	Pseudonyms gestation^a^	Parent^b^	Infant^c^
Evelyn, Baby	Age: 43 years	25 months’ old	Helen,	Age: 30 years	24 months’ old
Jenny	Home	Hospital stay:	Baby Daisy	Home	Hospital stay:
28 weeks	Full-time	12 weeks	28 weeks	Part-time	9 weeks 6 days
Maria, Baby	Age: 36 years	12 months’ old	Mia,	Age: 32 years	22 months’ old
Tommy	University	Hospital stay:	Baby Layla	University	Hospital stay:
30 weeks	Part-time	7 weeks	31 weeks	Full-time	6 weeks
Jill,	Age: 34 years	19 months’ old	Jane,	Age: 35 years	19 months’ old
Baby David	University	Hospital stay:	Baby Liam	University	Hospital stay:
32 weeks	Full-time	3 weeks 4 days	32 weeks	Full-time	4 weeks
Lauren,	Age: 32 years	17 months’ old	Kate,	Age: 30 years	29 months’ old
Baby Nancy	Home	Hospital stay:	Baby Megan	Home	Hospital stay:
28 weeks	Full-time	6 weeks	31 weeks	On leave	6 weeks
Lisa,	Age: 23 years	14 months’ old	Eleanor,	Age: 31 years	12 months’ old
Baby Alfie	University	Hospital stay:	Baby Eric	University	Hospital stay:
29 weeks	Part-time	7 weeks	32 weeks	Full-time	5 weeks
Jess,	Age: 35 years	13 months’ old	Ava,	Age: 22 years	21 months’ old
Baby Hugh	Home	Hospital stay:	Baby Ben	University	Hospital stay:
29 weeks	On leave	16 weeks 1 day	28 weeks	Student	12 weeks

Kate also had a 3-month-old infant born full-term; Ava was 24 weeks pregnant.

All ppts partners were working full-time. Ppts employment status (full-time, part-time, leave, student) and interview setting (home, university) is listed.

Infants’ ages were rounded up to the next month if their age included >15 days (e.g., 12 months 16 days).

### Theme 1—Emotional Impact

#### Distress

Within this subtheme, participants described experiences of trauma, shock, ongoing suffering, and feelings of self-blame. These emotions were particularly described as occurring during their time in the NICU.

I was standing at the window, and I watched my partner […] drive away as I had to stay in hospital, and I just cried… [the nurses] brought me a cup of tea and then just left me […] while my world was falling around them” (Lauren)

Most participants spoke of memories of leaving their infants in the hospital, which brought heightened distress that has persisted over time; “saying goodbye, I don’t think that is ever going to leave me just how hard it was to leave him in the hospital […], it’s just awful” (Jill). In addition, participants mentioned specific symptoms linked to a trauma response, such as flashbacks and recurring nightmares; “like every night you know [I had] a visual nightmare where I would wake up with the sweat and the tears and was up and standing around at her funeral” (Lauren).

There was a felt sense of responsibility for some participants regarding the health of their infant which they associated with feelings of distress; participants felt guilty of the harm they believed to have caused them and wondered what they had done. Participants also described finding it difficult to forgive themselves due to the self-blame they felt; “I think I’ll never fully forgive myself and I’ll always fully think there was something I did or didn’t do, that will always be at the back of my mind” (Jane).

I’ve felt like the whole thing was my fault […], so the first time that I held her […] I actually said to her ‘I’m sorry’ [crying] ‘cause I felt it was my fault, because it was my body that rejected the placenta and everything else (Mia)

Distress was also mentioned regarding the medical environment, such as unhelpful staff responses, perception of the environment as intrusive, and medical procedures occurring quickly.

She took me to about three other machines and then she got the consultant and he literally straight away said ‘yep, this needs to come out’ […]so he was delivered that night at twenty to ten by emergency section and I really wasn’t prepared for it” (Jess)

#### Feeling Unprepared and Fearful

This subtheme was linked to participants initially feeling unprepared for the experience and feeling fearful of what may happen to their infants in the present and future; “I think it was absolute terror whenever I found out that she was coming early it was like, I’m not ready for this, I have no clothes, I have no cot” (Mia).

Having an infant born prematurely caused much fear in participants regarding interacting with the infant and due to future thoughts of the potential difficulties that the infant may have; “I suppose when Megan was growing up, we were always worrying ‘would she meet this milestone, would she be developmentally okay?’” (Kate).

I remember the lady squirting hand sanitiser into my hand and saying, reach in and touch your baby, [..] and I said ‘no I can’t, I’m going to hurt her’ and I remember being frightened because she was so tiny (Helen)

#### Feeling Different

Participants described feeling different from their peers and feeling unable to connect with parents of full-term infants.

I got into arguments with a lot of people, like my mum and my sister […] just after he was born and it was because I think ‘you don’t understand what I’m going through, you don’t know what it’s like’. I know my sister has kids but it’s not the same (Jane)

**Figure 3.**
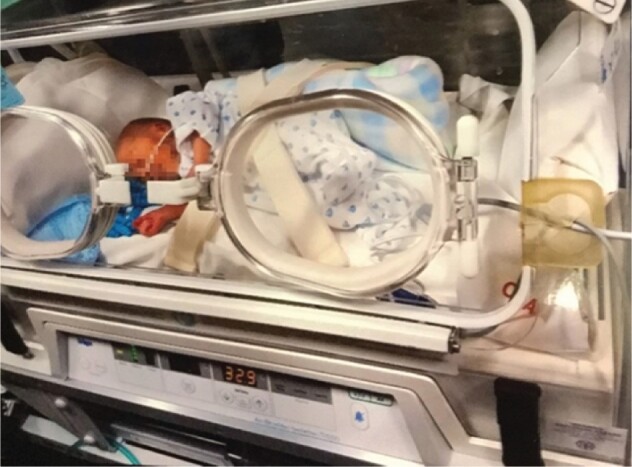
Photograph brought by participant Jill.

Feeling different was linked to loss. Participants had preformed ideals of what parenthood would be like, which they felt they were deprived of and had lost; “this [photograph] is his first car ride. So obviously whenever you think of your baby coming home you think your first car ride is going to be in your car seat... His was in an ambulance” (Jill; see Figure 3).

New Year came, and I collapsed in a heap and I was just like ‘this is not how our new year was meant to go. It’s our first year married, a baby coming’ [crying] we were meant to have so much hope (Lauren)

Participants realized that their life was different from where they thought they would be once they had a new-born. This sense of difference created a dichotomy between the present situation and what could have been, contributing to feelings of being different.

What I most looked forward to when I got home, was to be able to sit in the house in my pyjamas instead of getting up, getting ready, and going to hospital… so I feel like my whole maternity was completely different from a full-term baby (Lisa)

#### Frailty of Life and Well-being

Within this subtheme, participants described experiences where they were directly faced with the medical conditions of their infant and associated these experiences with feeling fearful. They also described experiences of anticipating their infants’ health deteriorating and their potential death; “I got [my partner] to take a video because I thought ‘if she passed away, at least I would have seen her move and be alive’” (Helen).

Participants hoped for a positive outcome but reported having constant reminders of the vulnerability of their infants. Participants also described their own physical and psychological frailty; “I was very sick after Megan was born. I had to have the drip on because they thought I was going to have seizures, so I didn’t get to see her for a full 24 hours ” (Kate). Although none of the parents in the sample were attending mental health services, several of them described believing they had suffered from mental health problems such as postnatal depression, post-traumatic stress disorder, or an anxiety disorder;

I feel like even now, I’m just getting back to who I was before Layla was born, although I was never diagnosed with any post-natal depression or anything like that, I think there was an underlying thing somewhere within me (Mia)

### Theme 2—Searching for Parent Identity

#### Not Feeling Like a Parent

Participants described an initial sense of not feeling like they were parents while in hospital due to the dependence on the medical system to keep their infants alive and feeling a loss of their parenting role. This was also related to not being responsible for performing basic care tasks and feeling like they were unable to initially strengthen their emotional bond with their infant. Participants contrasted their actual lived experience to what they had previously envisioned and hoped for; “I felt like I was still pregnant, but I wasn’t carrying him. The incubator is like my belly. I just left him off somewhere to be, to do the rest of the job, but I didn’t feel like I was a parent” (Ava).

**Figure 4.**
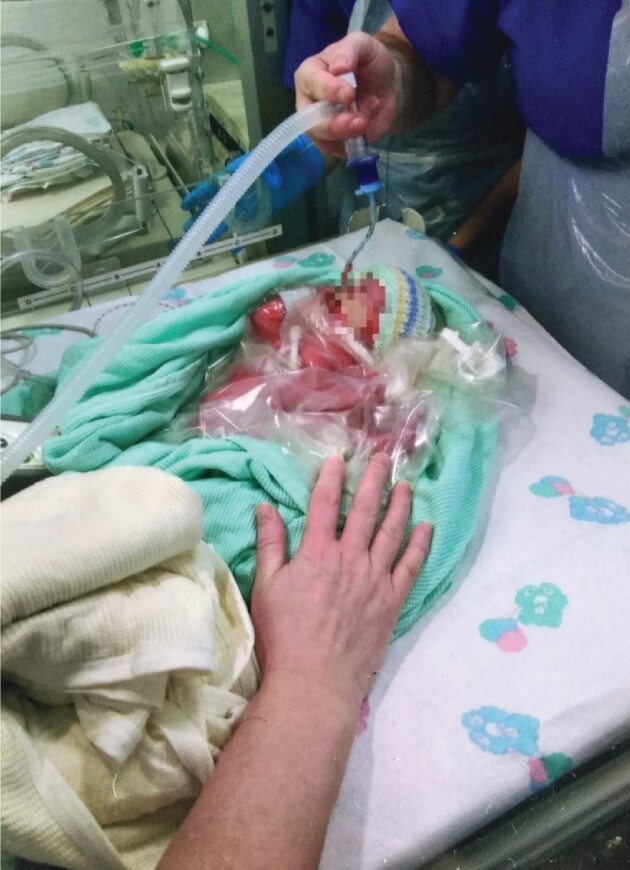
Photograph brought by participant Lauren.

They’re keeping her alive and warm. She’s wrapped in bubble wrap and a hat that we didn’t give her… there’s nothing of [my partner] and I in that picture - that’s not even one of our hands… she’s now separate from me, she’s just been taken out of my tummy and taken away from me - that’s what that picture means to me (Lauren; see Figure 4)

#### Regaining Parenting Role

Over the course of time, participants described instances of how they navigated regaining their parenting role and identity through engaging in basic care tasks, strengthening their relationship with their infants, and making up for the time lost when bonding with them. Participants described continually learning new skills as time went by and adapting to the needs of their infant. This process began in the NICU for most participants and was strengthened following discharge, as the dyad entered the home environment; “Changing his first nappies was through the wee holes in the incubator so I had to learn to adapt to an incubator to change his nappy rather than just actually learn how to put on a nappy” (Jill).

Once they are in the cot then the next step is home, so it felt like we took a real leap that day ‘cause she didn’t have her monitors. […] I could lift her out of the cot, hold her, nurse her… it just felt so much more natural’ (Mia)

During this shift from not feeling like a parent to regaining one’s parent identity, participants also reported seeking ways to establish an emotional connection and to make up for lost time.

I just thought ‘I didn’t get to hold this baby for six weeks so I’m going to make up for all these cuddles because before you know it, she’ll be walking, and I’ll not be able to hold her all the time’ (Kate)

The concept of returning to normality appeared across all transcripts. This involved reaching milestones such as leaving hospital, but also participants asserting their needs to establish a normative bonding experience to regain their parenting role.

This is just me and my baby and I’m just going to sit here and hold him and that’s okay […] it was the realisation of that, and I suppose just really trying to get to know him, getting our independence and not obsessing about being in a routine (Maria)

#### A Different Kind of Parenting

In this subtheme, participants reflected on how they adjusted to parenting a premature infant and spoke of how their experience was different from parents of full-term infants. They described themselves as engaging more in overcautious behaviors to protect their infants from illness and relapse.

[Other mummies] are not as obsessed with sterilising and cleaning. I suppose I had been in that sort of institutionalised environment - I was so into sterilising, infection control, all the things I suppose other preemie mummies were (Kate)

Participants described the impact the NICU environment and hospital protocols had on their parenting. Their parenting experiences upon birth began with an initial dependence on the medical equipment to keep their infants alive within hospital. Participants also spoke of the hospital medical routine and protocols dominating their home life; “there were a lot more appointments, there was a lot more check-ups, and she came home on medication… so there was a lot more that I had to do” (Evelyn).

There are lots of machines and lots of beeping and lots of things going on, but it’s so quiet and it’s almost like there’s a fear you know even though all the parents are there and it’s all their children, but there seems to be almost this fear and you just sit and you stare and it’s very strange (Jane)

### Theme 3—Moving Beyond Adversity

#### Hopeful Moments

In NICU, there was an underlying fear of infants becoming progressively ill or dying. Therefore, participants rejoiced when their infants reached milestones, as it represented progression and hope. These instances often began with participants realizing that their infant was born alive and being thankful for this. Paying attention to milestones reached within the NICU, helped participants feel closer to being discharged and embracing normality in their parenting. These milestones tended to be related to specific aspects of the infants’ medical care and physical well-being.

There was so many small steps but they were happening every hour every day, like his feeds, if a feed increased, that was amazing, you were focused on all the tiny milestones, like his weight going up by a couple of grams, so I just took it day by day (Lisa)

**Figure 5.**
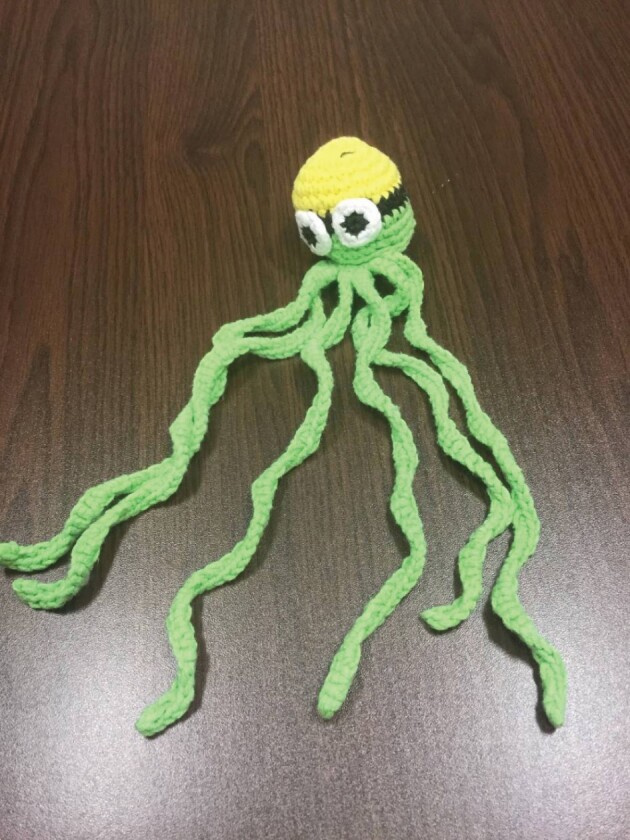
Photograph brought by participant Eleanor.

We used this [photograph] as a progress picture…one of the things I liked was when it was in the cot beside him - it was nearly the same size as him [laughs] and now he has pictures of it nearly every month with it sitting beside him. You can just see how much bigger he is and healthier (Eleanor; see Figure 5)

As described in the *Emotional Impact* theme, the underlying fears that participants communicated were of their infants relapsing due to illness or being behind their peers developmentally. Thus, the instances that instilled hope postdischarge related to meeting developmental milestones.

When he did meet [a developmental milestone], it was like ‘yes, this is amazing’. You are so proud and so happy, it’s almost like ‘tick, there’s another one off the list […] he might be doing it a little bit later than we had planned but he’s doing it and that’s okay (Jane)

Participants depicted their infants as having strength which gave them comfort that they would continue to thrive and survive; “I just think of her journey and what she’s came through and I’m like ‘how amazing’ as an adult we […] fall sick we just lie there, whereas these babies fight, and these babies are just incredible” (Helen).

#### Self-Care and Growth

Within this subtheme, participants described natural processes where they accepted the situation over time, learnt to deal with their emotions, and reinterpreted their experience; “I just got to a point I was like ‘d’you know what, I couldn’t have done anything different, like he’s here now and he’s healthy just it’s not my fault’” (Lisa).

Even a year ago if you had of said ‘tell me your story’ I would have broken down in tears halfway through and couldn’t have told you anymore, but I think I have dealt with much more and I have battled through it, and I have lived it […]it’s a raw emotion still but I’ve learnt how to manage those emotions you know? I don’t think you would ever get over it in the sense that it is just something that happened (Helen)

Examples of actions that helped participants, or advice they would suggest to other parents were also discussed as part of the learning and growth process. These included accepting the help of others, having more confidence in medical staff expertise, taking each day as it comes, not engaging in thoughts of self-blame, and keeping self-informed about medical procedures; “take the help that you can get, listen to people, and never ever blame yourself, ‘cause that’s probably what ate me for so long’” (Mia). Participants also recognized service improvement areas. One participant spoke of wanting more support for parents when dealing with the experience of leaving their infants each night.

You are never gonna feel fine leaving them, but I think just if somebody sort of came to you and said you know ‘this is normal to be feeling like this’ you know ‘try doing this’ or even just get you prepared for that (Jill).

#### Interpersonal Support

Interpersonal support reflects the impact that other people had on helping participants to cope and overcome adversity. Participants described specific interactions with staff members which helped them while in hospital such as reassurance provided, explaining medical terminology, prioritizing their emotional well-being, and responding to their concerns. Participants also described specific interactions with those outside of the immediate medical care (e.g., agencies, family, friends), which helped them cope better and move beyond adversity: “having somebody to talk to who’s also had a premature baby, on a day-to-day basis about various things has been really really lovely, so I value that because I think I would have really struggled had I not had that” (Maria).

## Discussion

Overall, when recounting their experiences of having an infant born premature, participants described moving from a state of emotional distress to a state of growth and acceptance. All participants in the study reported initially feeling distressed by the experience and the hospital environment. As depicted in existing findings, participants described feeling fearful, shocked, experiencing post-traumatic stress symptoms, and that they were to blame (Al Maghaireh et al., 2016; Carter et al., 2007; Helle et al., 2018). Similar to findings reported by Lindberg & Öhrling (2008), the majority of participants described the separation from their infants as being the most stressful part of their experience. This separation has been described by mothers as a barrier to their initial maternal identity development (Rossman et al., 2015). Upon birth, parents of premature infants are faced with factors that can contribute to ongoing stress such as being faced with the potential death of their infant, a fast-paced and often traumatic delivery, and exposure to an ongoing state of uncertainty regarding their infant’s development. In the subtheme of “self-care and growth,” participants described thoughts they developed over time to help them reappraise their situation and find strength from it. This is in line with findings that parents of children who survive their time in the NICU show higher levels of post-traumatic growth postdischarge and that this construct is impacted by parents’ positively reinterpreting a situation (Aftyka et al., 2017). Spinelli et al.’s (2016) description of the transition to motherhood as being suspended in time between past ideals, an unknown future, and present worries whilst parents are in the NICU was evident across transcripts. Factors that aided participants were reaching milestones and reinterpreting their experience over time. Reaching milestones both in hospital and following discharge appeared to help participants feel confident in their infant’s resilience and relayed a sense of progression to normality as is described in the subtheme “regaining parenting role.” In their model of parental progression, Hutchinson et al. (2012) describe that upon discharge from the NICU, parents become more autonomous and begin to define themselves psychologically as parents. Similarly, Jackson et al. (2003) describe parents moving to stages of confidence and familiarity in their parenting roles postdischarge. Across interviews, participants described a variety of actions that they undertook to regain their parenting role and to ease the impact of their distressing emotions. These ranged from finding moments at home to connect with their infant (i.e., making up for lost experiences within the NICU), focusing on how to support their infant reach developmental milestones, and maintaining parts of the hospital routine at home to help with their infant’s transition. Participants described feeling a stronger bond with their infant the more basic and emotional care tasks they engaged in whilst in the NICU and beyond. For some participants, it was only prior to discharge that they started to feel like parents, and they embraced opportunities to spend time with their infants alone.

Participants also described moving from thoughts and emotions of not feeling like a parent to adjusting to a different kind of parenting. The difference was in relation to comparisons made with parents of full-term infants and prior expectations about parenting. Parents of premature infants described themselves as initially being preoccupied with medical routines and appointments, having clean home environments, and not wanting to expose their infants to outside settings. Over time, they described embracing the difference and returning to normality. This process seemed to occur as their infant became older, less dependent on the medical system, and as parents became less fearful of illness. These findings are consistent with the literature on parents’ regaining confidence following discharge. Berman et al. (2019) state that parents of hospitalized infants report wanting more emotional support within the NICU and postdischarge. Some participants communicated the importance that healthcare professionals, family, other NICU parents, and community groups had on their growth process and ability to cope. There is a growing body of evidence that hospital or community-based peer support is of benefit to parents of infants hospitalized in the NICU (Hall et al., 2015).

### Strengths and Limitations

Interview guides and the order of questions can influence responses and subsequent analysis within qualitative research. Results showed a sense of a progression or journey that participants went through. This may have reflected the nature of the interview questions, which began with the birth story and ended with questions on current parenting experiences. This potential limitation was addressed through asking open-ended questions during the interview. Additionally, the research did not capture the experience from both parental perspectives and data were not collected on participants' racial and ethnic representation, potentially leading to a misrepresentation of the diverse kinds of parents living in Northern Ireland who experience a premature birth. All parents that participated were first time mothers and therefore the research did not capture being an experienced parent with a premature infant. These limitations may have been due to recruitment through social media and the kind of parent that engages with these mediums for information (e.g., first time parents, primarily mothers, access to technology). Notwithstanding these limitations, the sample was more homogenous than other studies in this area. Qualitative studies within this population have tended not to be homogenous and have comprised samples where individuals vary greatly on specific factors, including infants from wide ranging gestational ages and parents being interviewed at different timeframes since their premature birth experience (Kantrowitz-Gordon et al., 2016; Karatzias et al., 2007; Spinelli et al., 2016). Although qualitative research does not aim to generalize findings to the wider population, but to provide a rich understanding of people’s experience (Braun & Clarke, 2013; Polit & Beck, 2010), it is helpful for studies to have a specific group of participants to develop a general understanding about that group, stimulate thinking on the factors effecting that group, and inform further research and clinical considerations. The inclusion of a visual approach to data collection was a strength in the present study. Other types of stimuli that could have been considered include bringing specific objects to the interview related to the premature birth or auditory stimuli such as lullabies or songs associated with each participant’s parenting journey. Most participants reported finding the visual approach helpful and that it aided them to provide rich descriptions of their experiences. Participants also described that choosing photographs before the interview prepared them emotionally and aided reflection.

### Clinical Implications and Future Directions

Across the interviews, participants described emotional difficulties they experienced regarding their parent identity and infant’s prematurity and provided ideas on how their transition to parenthood in the NICU and beyond could be better supported. Along with existing studies highlighting emotional distress within similar samples, this supports the need for psychological interventions to be imbedded in hospital settings. Parent support interventions in the NICU could be delivered in the form of psycho-educational programs and psychological therapy, which have shown their effectiveness in relieving psychological distress in this population (Franck & O’Brien, 2019; Hall et al., 2015; Jotzo & Poets, 2005; Puthussery et al., 2018; Suttora et al., 2014). Participants in this study identified themselves as not currently seeking or attending mental health services, however, based on their reported emotional experiences following discharge, it may have been beneficial to have had access to psychological intervention at discharge. Thus, parent support interventions could be delivered in the community through psycho-educative and ongoing individual/group modalities. These interventions could be informed by existing literature and the findings of the present study and used as a medium for parents to reflect on their experiences and understand the range of emotions that exist in this population.

The present study revealed that participants did not feel like parents initially and wanted to be more involved in the care of their infants. Parent-partnered care models such as Family Integrated Care aim to train parents to be more involved in their infants’ care through being part of the NICU care team (Franck & O’Brien, 2019). This model has shown effectiveness in reducing levels of stress and anxiety in parents and has shown positive clinical infant outcomes (O’Brien et al., 2018). Participants in this study described adjusting over time to a different kind of parenting once their infants were discharged from hospital. Specialist community interventions focused on the evolving parent–infant relationship using an attachment perspective may also be of benefit to parents of infants born prematurely. As the study did not compare the identity development of parents of premature infants versus those of full-term infants, further research could focus on the impact that having an infant born premature has on one’s identity and feeling of being a parent and whether the differences persist over time. Considering the findings within the “moving beyond adversity” theme, further research using the construct of post-traumatic growth within this population would help to better understand the process of acceptance and to inform psychological interventions. As there are often worries in this parent population regarding the effects of prematurity on scholastic abilities, and meeting developmental milestones (Blackburn & Harvey, 2020), future research could examine parental experiences of growth/acceptance and the emotional impact of the perceived effects of prematurity as their infants’ progress through toddlerhood and school age.

## Funding

None to disclose.
